# Fitting *C* ^2^ Continuous Parametric Surfaces to Frontiers Delimiting Physiologic Structures

**DOI:** 10.1155/2014/278479

**Published:** 2014-03-24

**Authors:** Jason D. Bayer, Matthew Epstein, Jacques Beaumont

**Affiliations:** ^1^L'Institut de Rythmologie et Modélisation Cardiaque, Université de Bordeaux, 166 Cours de l'Argonne, 33000 Bordeaux, France; ^2^Department of Bioengineering, Binghamton University, P.O. Box 6000, Binghamton, NY 13902, USA; ^3^Department of Pharmacology, SUNY Upstate Medical University, 3135 Weiskotten Hall, 750 East Adams Street, Syracuse, NY 13210, USA

## Abstract

We present a technique to fit *C* ^2^ continuous parametric surfaces to scattered geometric data points forming frontiers delimiting physiologic structures in segmented images. Such mathematical representation is interesting because it facilitates a large number of operations in modeling. While the fitting of *C* ^2^ continuous parametric curves to scattered geometric data points is quite trivial, the fitting of *C* ^2^ continuous parametric surfaces is not. The difficulty comes from the fact that each scattered data point should be assigned a unique parametric coordinate, and the fit is quite sensitive to their distribution on the parametric plane. We present a new approach where a polygonal (quadrilateral or triangular) surface is extracted from the segmented image. This surface is subsequently projected onto a parametric plane in a manner to ensure a one-to-one mapping. The resulting polygonal mesh is then regularized for area and edge length. Finally, from this point, surface fitting is relatively trivial. The novelty of our approach lies in the regularization of the polygonal mesh. Process performance is assessed with the reconstruction of a geometric model of mouse heart ventricles from a computerized tomography scan. Our results show an excellent reproduction of the geometric data with surfaces that are *C* ^2^ continuous.

## 1. Introduction

High-resolution* in vivo* imaging has become an essential tool for the practice of modern day medicine. As a matter of fact, hospitals have upgraded their infrastructure to acquire and visualize terabytes of digital medical data and to make these data readily available to the clinician. Prompted by this success, more and more clinicians introduce cutting edge technology in diagnosis. We can say we witnessed the rise of what we have come to term computational medicine.

In this trend, among various options to capitalize on the wealth of medical data is the simulation of biophysical processes. This option is particularly appealing as it equips the clinician with advanced means to make elaborate interpretations from data collected non- or semi-invasively, which can potentially lead to a better understanding of the molecular mechanisms of diseases, detection of trends in a population, and risk/benefit analysis of various therapeutic options.

A major facet of computational medicine is the reconstruction of image-based computer models of physiologic structures, for example, tissue, organs, and limbs, in order to effectively interface with the clinician. In cardiology, we count a number of contributions in heart model reconstruction [1–5] that enable us to be optimistic in terms of practical clinical applications in the near future. Clearly, the same applies to neuroscience [6–8].

A very important step in the reconstruction of image-based models is the mathematical description of surfaces bounding the physiologic structure of interest. Here our goal is to generate a compact *C* ^2^ continuous representation of surfaces. We elect to use dimension 3 (3 physical coordinates) and codimension 2 (2 parametric coordinates) parametric B-splines. Such surfaces have a number of advantages for modeling; that is, they are compact and facilitate the calculation of areas, volumes, curvatures, crests, tangent planes, and many other operations which are useful by themselves and essential in several modeling phases, like mesh generation, application of boundary conditions, and coupling with several field equations.

While the fitting of curves to scattered geometric data points is quite trivial, the fitting of surfaces to scattered geometric data points is not. Surface fitting is problematic because a unique parametric coordinate should be assigned to each geometric data point, and the precision of the fit is quite sensitive to the distribution of these parametric coordinates. To our knowledge, there are no documented techniques to assign unique parametric coordinates and to distribute them in a manner that guarantees a precise fit.

There has been significant work on this question. So far two different approaches have been proposed. In the first approach, the surface in question is the zero level set of a three-dimensional function that is fitted to the data. This avoids the mapping of the geometric data onto a parametric plane since the function in question is defined in the same space as the original image. Most algorithms have been implemented with polygonal surfaces, but could also be implemented with B-splines [9].

The second approach, deformable models, deforms an initial template model until it matches the target geometric data points. The technique initially introduced by Kass et al. [10] has been given considerable attention. The contributions of McInermey and Trezopouloz [11, 12] and Yoo [13] are particularly remarkable to the subject. The template model can be defined with a polygonal or parametric B-Spline surface, and various rules can be used to guide the deformations, for example, intensity gradient, point-to-surface signed distance, mechanical properties of the surface, inertia in displacement, and others like energy, which depends on the physics of the problem. In a version of this approach, the conventional rules to guide deformation (e.g., intensity gradient) are replaced by a function based on the comparison of pixel intensity with the average intensity of pixels inside and outside regions delimited by the displaced boundary [14]. This version is particularly robust (no manual assistance required) in the presence of noise and low contrast. Although this advantage comes at the expense of an additional computational load due operations performed on all image pixels at each iteration, which may be acceptable in a number of cases. Finally, see Park et al. [15] for a recent extensive application, and the review of image segmentation by Ma et al. [16] for a summary of applications.

Here we propose a different approach, that is, a direct fit of codimension 2 parametric B-splines to a polygonal surface extracted from a segmented image. After its extraction, the polygonal surface is projected onto a parametric plane. The projection preserves vertex connectivity and ensures a one-to-one mapping between the physical and parametric spaces. Afterward, the projected polygonal surface is regularized for polygonal area and edge length. The spatial variation in polygonal area and edge length is quantified by a function, the minimization of which governs the regularization. Finally, from this point parametric surface fitting, constrained or not, is quite trivial. The main advantages of our technique are its accuracy and computational simplicity.

The method is applied to an X-ray computerized tomography scan of a mouse heart. The results show that *C* ^2^ continuous parametric surfaces with only 9 × 9 degrees of freedom can accurately fit convoluted geometries like the endocardial surface of the heart.

## 2. Methods

### 2.1. Acquisition of Test Medical Imaging Data

An adult mouse heart is excised, Langendorff perfused, fixed in diastole, and then embedded in vegetal grease. It is subsequently imaged with an X-ray laboratory scanner (SkyScan) at 7.5 *μ*m resolution. A computerized tomography (CT) scan is generated with 720 projections. Each projection is integrated over 1.8 s. The background is subtracted; the signal is compensated for fluctuations and converted on a logarithmic scale. The signal is finally postprocessed with a modified Feldkamp tomographic reconstruction algorithm [17]. See Methods in Bayer et al. [18] for more details on the specimen preparation and CT imaging.

The same heart is also imaged for tissue microstructure with a laser scanning confocal microscope. To this end, the heart is sliced every 20 *μ*m along its short axis with a vibrotome. Each slice is stained for myofibrils with rhodamine phalloidin. They are subsequently imaged with a Zeiss 510 META confocal microscope. The images are acquired on optical planes separated by 5 *μ*m. Further details on staining and imaging are given in Slamani et al. [19]. Acquisition at this high resolution requires assembling the images in order to appropriately visualize the physiologic structure, which we perform with a Fourier transform [19, 20].

### 2.2. Polygonal Surfaces Extraction

The CT data is composed of gray scale images (pixel intensities 0–255) stacked along the long axis of the heart. They comprise a total of 860 planes. The contrast is excellent, and the edges of the heart cavities, as well as the epicardium, are simply delineated with thresholding. Specifically, all voxels crossing a threshold intensity of 100 are labeled as edge voxels. The external faces of these voxels (quads) constitute the polygonal surface we project onto the parametric plane.

When we need to extract a triangulated surface for visualization purposes, or because a very high precision is required, we extract it from the boundary voxels with the marching cube algorithm of Lorensen and Cline [21], along with modifications introduced by Lewiner et al. [22].

We denote the set of vertices and triangles or quadrilaterals generated from the surface extraction by *V*(*x*, *y*, *z*) and *T*(*V*) or *Q*(*V*), respectively. Then the inverse connectivity vertices-triangles *V*(*T*) or vertices-quads *V*(*Q*) is generated. Finally, a graph of the nodes *C*(*V*) (table of vertices adjacent to a vertex) is readily generated from the connectivity table *T*(*V*) or *Q*(*V*).

### 2.3. Polygonal Surface Projection onto a Parametric Plane

Direct parametric surface fitting requires that we first assign a unique parametric coordinate to each of the *N* vertices of *V*(*x*, *y*, *z*). We achieve this by projecting the polygonal surface onto a *u* − *v* parametric plane; *u* ∈ [*u*_min⁡_, *u*_max⁡_], *v* ∈ [*v*_min⁡_, *v*_max⁡_]. The projection assigns parametric coordinates *𝒱*(*u*, *v*) to each vertex in *V*(*x*, *y*, *z*). We then refer to the projected surface by *T*(*𝒱*) or *Q*(*𝒱*). To project the polygonal surfaces, the user selects a region to map onto the polygonal surface, which is delimited by 4 anchor points joined by 4 segments. Each of the 4 anchor points are mapped to one corner of the parametric plane, and the vertices on the segments joining them are equidistributed along the edges Γ ∈ [Γ_*a*_, Γ_*b*_, Γ_*c*_, Γ_*d*_], that we label *a* − *d* while following a boolean order, that is, *u*_min⁡_, *u*_max⁡_, *v*_min⁡_, *v*_max⁡_. We denote the set of indices of the vertices mapped to the edges of the parametric plane by *g*. The region inside the 4 segments is mapped onto the parametric plane. The rule employed to perform a one-to-one mapping is deduced from the function

(1) $$\begin{array}{c} \vartheta \left(u,v\right)=\overset{N-1}{\underset{n=0}{\sum }}\;\overset{M_{n}-1}{\underset{m=0}{\sum }}{\left(u_{n}-u_{m}^{\left(n\right)}\right)}^{2}+{\left(v_{n}-v_{m}^{\left(n\right)}\right)}^{2}, \end{array}$$

where (**u**, **v**) denote the *u* and *v* parametric coordinates of the vertices of *V*(*x*, *y*, *z*). Throughout this paper we denote vectors and matrices by lower- and uppercase bold letters, respectively. Therefore, the parameter *M*_*n*_ is the number of adjacent vertices to vertex *n* with coordinates (*u*_*n*_, *v*_*n*_). The *M*_*n*_ adjacent vertices to *u*_*n*_, *v*_*n*_ are denoted by *u*_*m*_^(*n*)^, *v*_*m*_^(*n*)^. These indices correspond to line *n* in the graph *C*(*V*).

The function *ϑ*(**u**, **v**) expresses for each vertex of  *V*(*x*, *y*, *z*) the sum of squared distance to its neighbors. The *Min*⁡{*ϑ*(**u**, **v**)} with respect to (**u**, **v**),

(2) ∂ϑ(u,v)∂uα=Mαuα−∑m=0Mα−1umα=0,∂ϑ(u,v)∂uα=Mαvα−∑m=0Mα−1−1vmα=0   ∀α∈[0,N−1],

leads to the matrix systems

(3) $$\begin{array}{c} A^{u}u=c^{\left(u\right)}\;\;A^{v}v=c^{\left(v\right)}. \end{array}$$

Since we fix the coordinates of the vertices along the edges of the parametric plane, the lines and columns of (3) corresponding to indices *g* are moved to the right hand side, which lead to the reduced matrix systems

(4) $$\begin{array}{c} {\hat{A}}^{u}\hat{u}={\hat{c}}^{\left(u\right)}\;\;{\hat{A}}^{v}\hat{v}={\hat{c}}^{\left(v\right)}. \end{array}$$

Since ${\hat{A}}^{u},\;\;{\hat{A}}^{v}$ are nonsingular, this matrix system generates a one-to-one mapping. The coordinates obtained in this manner correspond to a distribution where each vertex tends to be at an equal distance from all its immediate neighbors. This confers interesting properties to the map, but does not mean all points are equidistant from one another. This property is desirable at the expense of additional processing.

Note the particular structure of the matrices ${\hat{A}}^{u},\;\;{\hat{A}}^{v}$. First, they are sparse and the number of nonzero coefficients in each line is given by the number of neighbors in the parametric plane. The diagonal is *M*_*n*_, *n* ∈ [0, *N* − 1], *n*∋*g* and the off diagonal elements are −1. The matrices are diagonally dominant, and thus positive definite. Furthermore, since the coefficients are constant we need only to store in memory their diagonal. Such matrices have a small condition number and can be readily solved with a conjugate gradient method.

When the point cloud is really dense, we may sometimes need to postprocess the map to avoid a collision between vertices. If the bounding box of two segments intersects in the parametric plane, we replace the endpoint on each segment closest to the intersection by their intersection. Finally, when segments sharing one vertex are nearly parallel, we fuse them. Note that such postprocessing is necessary only when the point cloud is really dense. In most applications this can be avoided by decomposing the reconstruction.

### 2.4. Regularization of a Polygonal Mesh

While the mapping of the polygonal surface to the parametric plane is a one-to-one mapping, this does not guarantee the size of the polygons (triangles or quadrilaterals) to be regular. Irregularity in size dramatically affects the quality of the final parametric surface fit because areas that are extensively compressed in the parametric plane are given less weight, if not totally ignored, in the fit. This may be alleviated by increasing the number of elements of the final parametric surface, but this does not solve the problem.

We address this problem by regularizing the polygons of the mesh for area and edge length. To do so, we borrow a mesh regularization concept introduced by Jacquotte in [23]. The concept consists of adjusting the grid-point coordinates in a manner that minimizes polygonal element deformations with respect to a reference element.

First, we define a transformation or a mapping from the space *ξ* − *η* to the parametric space *u* − *v*,

(5) $$\begin{array}{c} 𝒯\left(\xi ,\eta \right)=\left[U\left(\xi ,\eta \right),V\left(\xi ,\eta \right)\right], \end{array}$$

where *U*(*ξ*, *η*), *V*(*ξ*, *η*) are two functions mapping the *ξ*, *η* coordinates of a reference element defined on *ξ*, *η* ∈ [−1,1] to the (*u*, *v*) coordinates of the parametric plane, respectively. The reader is referred to Appendix A for details regarding the construction of mapping functions for both quadrilateral and triangular meshes. The transformation *𝒯*(*ξ*, *η*) maps a reference quadrilateral to a mesh element according to solid mechanics, where the element deformation gradient is given by

(6) $$\begin{array}{c} \bar{\nabla }𝒯\left(\xi ,\eta \right)=\left[\begin{array}{cc} U_{\xi } & U_{\eta }\\ V_{\xi } & V_{\eta } \end{array}\right],\;\;\bar{\nabla }=\left[\frac{\partial }{\partial \xi },\frac{\partial }{\partial \eta }\right], \end{array}$$

and the subscripts indicate derivative with respect to this variable. Note from (6), the deformation gradient is also the Jacobian matrix *𝒥* of the mapping between the *ξ* − *η* and *u* − *v* spaces. This quantity is precisely what we wish to minimize; that is, a regularization is nothing else than a minimization of the variation in element deformation. However, performing this minimization over the *u* − *v* parametric domain is not trivial. For the function to reach the condition we are looking for, that is, *𝒱*(*u*, *v*) having polygons with quasi-equal areas and edges length in the parametric plane, the function should be convex with one minimum reflecting that condition. This implies our measure of deformation to be invariant of rotation, since rotation does not change area and edge length.

Jacquotte in [23] addressed this problem by minimizing the Matrix **H** = *𝒥* ^*T*^ *𝒥* with respect to three invariants. From this point, our approach differs from Jacquotte's approach since we find new parametric grid coordinates (**u**′, **v**′) satisfying,

(7) $$\begin{array}{c} \left(u^{\prime },v^{\prime }\right):\mathrm{Min}\left\{\overset{N_{e}-1}{\underset{e=0}{\sum }}{||{𝒥}^{e}-\frac{\text{Adj}\left({𝒥}^{e}\right)}{\left|{𝒥}^{e}\right|}||}_{F,\Omega_{e}}\right\}, \end{array}$$

where *N*_*e*_ is the number of elements in the polygonal surface, *𝒥* ^*e*^ is the Jacobian of element *e*, Adj{·} is the adjugate, |·| is the determinant, *Ω*_*e*_ is the domain of an element in the *ξ* − *η* plane, and ||·||_*F*_*Ω*__ is a modified Frobenius norm

(8) $$\begin{array}{c} {||\left(\cdot \right)||}_{F,\Omega }=\underset{i}{\sum }\;\underset{j}{\sum }\int_{\Omega }{\left[{\left(\cdot \right)}_{i,j}\left(\Omega \right)\right]}^{2}d\Omega , \end{array}$$

which needs to be introduced because the matrix coefficients are a function of (*ξ*, *η*). Recall, the rightmost term is nothing else than *𝒥* ^−1^. The motivation for this condition is that when the reference element is subjected only to a translation, *𝒥* = *I*. In this case *𝒥* = *𝒥* ^−1^, and condition (7) is strictly met. The modified Frobenius norm of *𝒥* − *𝒥* ^−1^ increases with scaling, and changes slightly with rotation. Thus, the minimization of (7) tends to keep all elements shapes closed to the reference one.

### 2.5. Constrained Parametric Surface Fitting

The final surface is represented with a codimension 2, dimension 3 parametric B-Spline. The parametric plane is subdivided into *N*_*bx*_ × *N*_*by*_ elements of equal size. This subdivision defines (*N*_*bx*_ + 1)×(*N*_*by*_ + 1) junction points that we term the control points. Functions *X*(*u*, *v*), *Y*(*u*, *v*), *Z*(*u*, *v*) mapping the *u* − *v* space to the *x* − *y* − *z* space, one for each of the (*x*, *y*, *z*) coordinates, respectively, are defined with an expansion of codimension 2 B-Splines, one B-spline *B*_*s*_(*u*, *v*) for each control point, “*s*” indexing them. To the control points defined in the parametric plane, we add a row and a column at each end so the elements bordering the parametric plane have the same degree of freedom as the elements inside the plane. Thus, the total number of control points is (*N*_*bx*_ + 3)×(*N*_*by*_ + 3). Parametric surface fitting consists in finding the coefficients *ζ* ^(*x*)^, *ζ* ^(*y*)^, *ζ* ^(*z*)^ of the expansion

(9) $$\begin{array}{c} X\left(u,v\right)=\overset{\bar{s}}{\underset{s=0}{\sum }}\zeta_{s}^{\left(x\right)}B_{s}\left(u,v\right),\\ Y\left(u,v\right)=\overset{\bar{s}}{\underset{s=0}{\sum }}\zeta_{s}^{\left(y\right)}B_{s}\left(u,v\right),\\ Z\left(u,v\right)=\overset{\bar{s}}{\underset{s=0}{\sum }}\zeta_{s}^{\left(z\right)}B_{s}\left(u,v\right), \end{array}$$

$\bar{s}=(N_{bx}+2)\times (N_{by}+2)$ such that the distance between the geometric data points and points on the surface with the same parametric coordinate is minimized.

The *B*_*s*_(*u*, *v*) are indexed according to a boolean order, *u*-coordinate first then the *v*-coordinate. They are built with a tensor product of elementary 1-D B-Splines,

(10) $$\begin{array}{c} B\left(u,v\right)=b\left(u\right)⊗b\left(v\right). \end{array}$$

At control point “*n*” of a one-dimensional axis, we define the elementary B-spline by

(11) $$\begin{array}{c} b{\left(u\right)}^{3}=\{\begin{array}{cc} b_{0}\left(u\right)=\frac{\left(1-3\bar{u}+3{\bar{u}}^{3}-{\bar{u}}^{3}\right)}{48}, & \\ \bar{u}=2u-\frac{\left(u_{n+2}+u_{n+1}\right)}{\left(u_{n+2}-u_{n+1}\right)}, & \\ \;\;\text{for}\;u_{n+1}<u>u_{n+2}, & \\ b_{1}\left(u\right)=\frac{\left(23-15\bar{u}-3{\bar{u}}^{2}+3{\bar{u}}^{3}\right)}{48}, & \\ \bar{u}=2u-\frac{\left(u_{n+1}+u_{n}\right)}{\left(u_{n+1}-u_{n}\right)}, & \\ \text{for}\;u_{n}<u>u_{n+1}, & \\ b_{2}\left(u\right)=\frac{\left(23+15\bar{u}-3{\bar{u}}^{2}-3bu^{3}\right)}{48}, & \\ \bar{u}=2u-\frac{\left(u_{n}+u_{n-1}\right)}{\left(u_{n}-u_{n-1}\right)}, & \\ \text{for}\;u_{n-1}<u>u_{n}, & \\ b_{3}\left(u\right)=\frac{\left(1+3\bar{u}+3{\bar{u}}^{2}+3{\bar{u}}^{3}\right)}{48}, & \\ \bar{u}=2u-\frac{\left(u_{n-1}+u_{n-2}\right)}{\left(u_{n-1}-u_{n-2}\right)}, & \\ \text{for}\;u_{n-2}<u>u_{n-1}, & \end{array} \end{array}$$

where it should be clear that the support (range where the function is nonnull) of an elementary 1D B-spline extends over 4 segments, and the support of a 2-D B-spline over 4 × 4 elements. The B-Spline (1-D or 2-D) and its first 2 derivatives vanish at the border of the support. This way any linear combination remains *C* ^2^ continuous. More information on B-splines can be found in Bartels et al. [24].

Least square fitting for this case is quite trivial. It consists to find **ζ** ^(*x*)^, **ζ** ^(*y*)^, **ζ** ^(*z*)^ that minimizes

(12) $$\begin{array}{c} {||x-X\left(u,v\right)||}_{2},\;\;{||y-Y\left(u,v\right)||}_{2},\\ {||z-Z(u,v)||}_{2}, \end{array}$$

with **x**, **y**, **z** being the vectors of *x*, *y*, *z* coordinates of the geometric data points. This leads to solving 3 matrix systems, each of small dimension. Excellent results were obtained with 9 × 9 elements. In addition, the matrices are sparse with nonnull coefficients in each row and are well conditioned as a result of the regularization. Thus, they can be readily solved with an iterative method like the Generalized Mimimal Residual (GMRES) [25, Chapter 6].

For practical reasons the fit is constrained in two ways. First, we impose a degree of stiffness to the surface. Second, we sometimes require the parametric surface to exactly match a predefined contour. After visual inspection a user may wish to limit the excursion of a surface in zones not having enough geometric data points. This can be done by imposing the surface to be more stiff. The second constrains allows to decompose of the reconstruction of a complex physiological structure. This version of our reconstruction technique does not allow imposing *C* ^1^ continuity at the junction between parametric surfaces, but this could be added by extending the treatment presented in Appendix B.

Stiffness is added by constraining the fit with curvature and twist. In this case the objective function to optimize the *x*-component becomes

(13) Θx=(1−λ)||x−X(u,v)||2 +λ∫Ωp(Xuu2+2Xuv2+Xvv2)dΩp,   λ∈[0,1],

*Ω*_*p*_: parametric plane, and similarly for the (*y*, *z*) components.

To clamp the parametric surface along any of its edges, we fit the contour with a codimension 1 B-spline. The codimension 1 B-spline should have exactly the same number of control points as the codimension 2 B-spline along the axis of the clamp. Also, the parametric segments should be exactly the same length as the sides of the quads of the codimension 2 B-splines along the axis of the clamp. To guarantee an exact clamp, we remove some coefficients from the expansion of the codimension 2 B-spline and fix them with algebraic relations that guarantee the parametric surface to match the codimension 1 B-spline along the clamped edge. Details of this calculation are given in Appendix B.

### 2.6. Distance Metrics

We assessed the performance of our fitting algorithm by measuring the distance between the target geometric data points and the model surface. For a data point “*n*” in *V*(*x*, *y*, *z*), the distance between this point and the model *D*_*n*_(*u*, *v*) is the closest distance from this point to the model surface. Determination of minimal distance by sweeping points on the parametric surface can be time consuming and even inaccurate since the surface can undergo significant excursion between the sampled data points. On the other hand, bicubic B-Splines are too complex for analytical determination of minimal distance.

Here we explore the flexibility of the parametric surface representation to tackle this problem. First, we seek the minimal distance along *u* = *constant* and *v* = *constant* coordinate lines, 4 in each direction on any element. The derivatives

(14) $$\begin{array}{c} \frac{\partial D_{n}^{2}\left(u,v\right)}{\partial u},\;\;\frac{\partial D_{n}^{2}\left(u,v\right)}{\partial v}, \end{array}$$

along a *v* or *u* coordinate line is a 5th degree polynomial. We find its zeros with an eigenvalue method [26]; that is, we write a companion matrix, the eigenvalues of which are the polynomes zeros. The eigenvalues in question are found with the method outlined in LAPACK [27, Section 2.4.5]. The companion matrix is transformed into an upper Hessenberg form, which is subsequently reduced to a tridiagonal form, the diagonal of which contains the eigenvalues. Our implementation exploits the sparsity pattern of the companion matrix which makes the root finding fast, but still accurate. *D*_*n*_(*u*, *v*) is evaluated on all roots of the 8 coordinate lines if the corresponding *u*, *v* coordinate is in the B-spline element. It is also evaluated on the 4-element vertices.

The search is further refined with a fixed point method, starting from the closest of the above distances *u*_*c*_, *v*_*c*_. The surface *D*_*n*_(*u*, *v*) is approximated (${\hat{D}}_{n}(u,v)$) at *u*_*c*_, *v*_*c*_, with

(15) D^n(u,v)=[(u−uc),(v−vc)]H[(u−uc)(v−vc)]+g[(u−uc)(v−vc)]+Dn(uc,vc),

where **H** and **g** are the Hessian and gradient of *D*_*n*_(*u*, *v*) at *u*_*c*_, *v*_*c*_. The minimum of (15) is found by solving the 2 × 2 matrix system expressing the minimum condition, (*u*, *v*)→(*u*_*c*_, *v*_*c*_), whose operation is repeated until |**g** | <*ϵ*, a predetermined threshold, or when the line segment joining the current minimum to the new one crosses an element edges. The final minimal distance is the minimum of the minimal distance over all elements.

## 3. Results

### 3.1. The Cardiac Imaging Data

**Figure 1:**
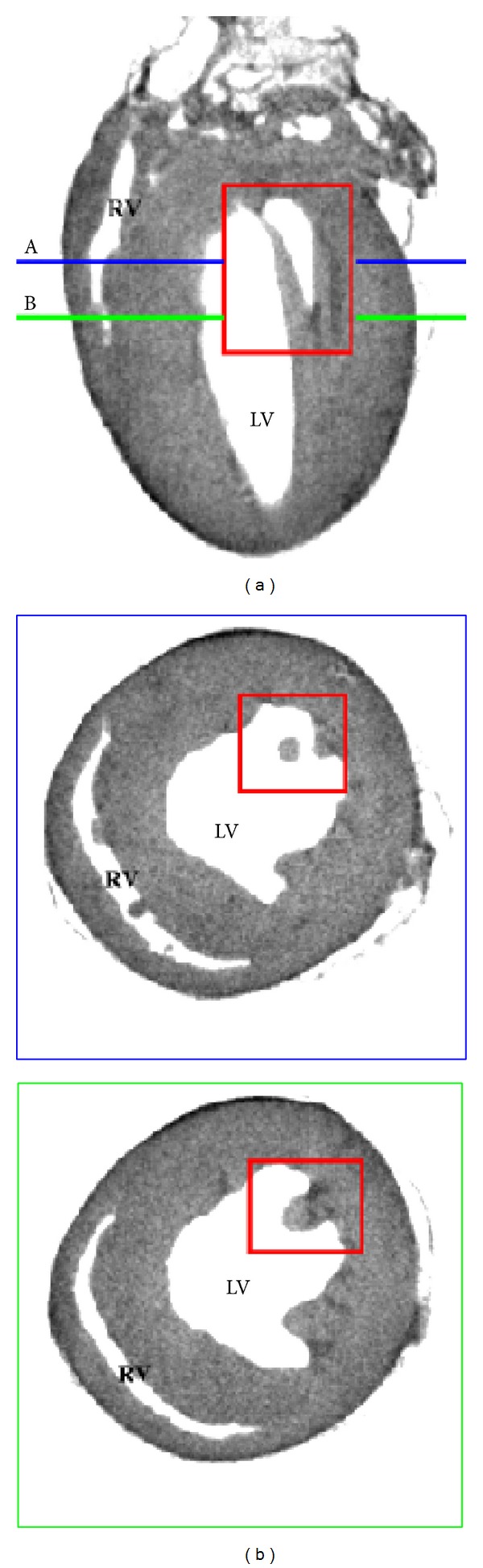
Computerized tomography scan of a mouse heart fixed in diastole. (a) Longitudinal section of the heart perpendicular to the septum. (b) Two sections along the short axis of the heart.

The test data set (Figure 1) is an X-ray computerized tomography scan of an excised mouse heart fixed in diastole. The data were collected on a laboratory microscanner (CT) at 7.5 *μ*m resolution. See the Methods for specimen preparation and data acquisition. Figure 1 shows three sections across this data set: one longitudinal section perpendicular to the septum and two cross sections along the short axis of the heart. The right and left ventricles are labeled RV and LV, respectively. The blue and green lines indicate the height of the section. The longitudinal section is 1.125 mm from the epicardial surface, and the two cross sections are 2.475 mm (blue) and 2.775 mm (green) from the atrial apex, respectively. The distance from the base to the ventricular apex is 7.050 mm, and the diameter at the base is 1.880 mm.

The longitudinal view exposes a papillary muscle (red rectangle) extending from the left endocardial surface to the aortic valve. The cross section at 2.775 mm shows the root of the papillary muscle. The other section at 2.475 mm clearly delineates a cross section of the papillary muscle which is detached from the endocardial surface. We believe papillary muscles play an important role in the initiation of abnormal beats since cardiac fiber changes rapidly in such region. Therefore, accurate modeling of these structures is a main motivation for the geometric modeling technique developed here.

Two other masses are visible in the cross section at 2.475 mm of the right ventricle. These are probably papillary muscles thorn during the heart preparation. The injection of grease, although done at relatively low pressure, may have damaged the fragile papillary muscles.

Finally, from this image the reader can appreciate the fact that contrast is excellent. No additional processing was performed after the tomographic reconstruction; still the edges of the cavity and epicardium are clearly visible.

### 3.2. Polygonal Surface Extraction

**Figure 2:**
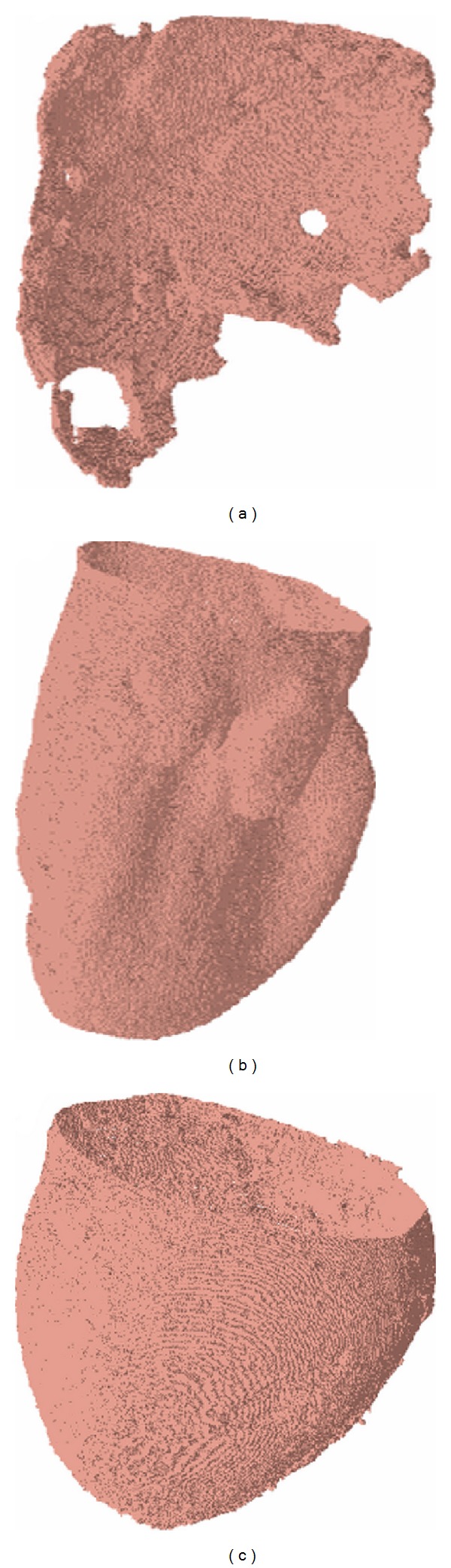
Polygonal surfaces delineating the right ventricular cavity (a) the left ventricular cavity (b), and the epicardium (c) of the mouse heart shown in Figure 1. The surfaces were extracted automatically by thresholding.

The ventricular cavities and epicardium of the heart CT scan of Figure 1 were delineated with a thresholding algorithm.  Figure 2 shows the external faces of hexahedra with vertices crossing the 100 gray level. This results in a brick wall texture, but, as shown in* constrained surface fitting* below, the polygonal surface becomes quite smooth when triangles are extracted with a marching cube algorithm [21, 28] from the hexahedra.

Figure 2(a) illustrates the practicality of geometric model reconstruction. The two holes in the edges of the right ventricular cavity are clearly artifacts. They appeared because we did not have any voxels crossing the prescribed threshold value. There are limits to CT, which could be compared to shadows in visible light. The reconstruction problem is partly a local operation and partly a global one. Local operations are needed to detect edges in general, but global information with a priori knowledge of the physiology is necessary to overcome limitations of local operations. Here the holes in the frontier of the RV cavity could be filled with user-assisted operations, which is time consuming. Alternatively, our approach prescribes rules to fit a smooth parametric surface on the polygonal surface. The RV displays this problem since the surfaces bounding the geometry are close to one another. However, for many problems including modeling and simulation, a smooth representation along this edge is quite adequate. Note that this problem could have potentially been eliminated by thresholding the intensity gradient instead of the absolute intensity value, or by using a more elaborate surface extraction method. However, at this stage the precision is sufficient to meet modeling needs.

As shown in Figure 2(b), when the edges of the structure imaged are not too close to one another, the surface can be accurately delineated. For example, we see very well the endocardial surface of the left ventricular cavity, even in the papillary muscle region despite the fact that it is quite convoluted.

Finally, the delineation of the epicardial surface is quite trivial. Thus, as long as the imaging modality offers good contrast and surfaces are not too close to one another, they can be delineated relatively accurately with thresholding.

### 3.3. Polygonal Surface Projection onto a Parametric Plane

**Figure 3:**
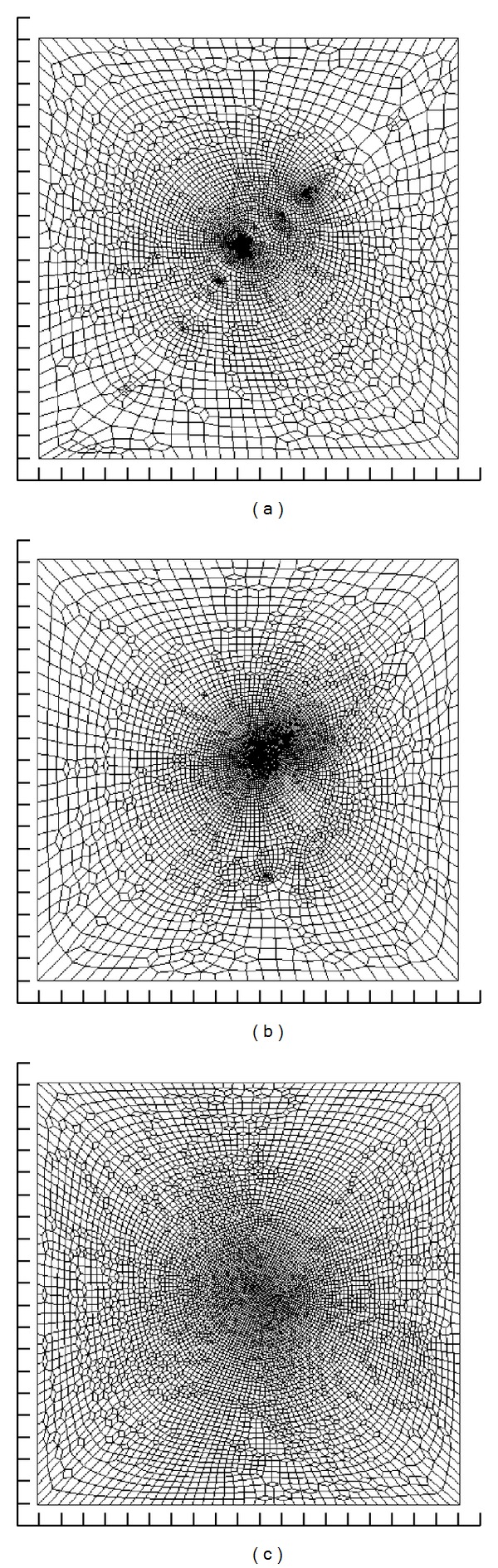
Polygonal surfaces (Figure 2), projected onto the (*u* − *v*) parametric plane. The surfaces delineate the endocardial surface of the right ventricular cavity (a), the endocadial surface of left ventricular cavity (b), and the epicardial surface (c).

Figure 3 shows *Q*(*𝒱*) obtained by projecting *Q*(*V*) of Figure 2 onto (*u* − *v*) parametric planes. Each parametric axis has 100 units, which is arbitrary. For the projections in Figure 3, line segments of *Q*(*𝒱*) do not intersect and a one-to-one mapping was achieved. The epicardial surface is relatively regular and, as a result, the polygonal mesh generated by the projection is also relatively regular. The quads are more dense around the center compared to the periphery, but the difference is not very large. This is in contrast to the RV and LV surfaces. In these 2 cases, the density of quads is much larger around the center. In addition, the RV surface has several epicenters. The difference in quad density between these epicenters and the periphery is quite large, which is typical for convoluted geometries.

The RV and LV polygonal meshes illustrate well the parametric surface fitting problem. When we fit a codimension 2 parametric B-spline, the *u* − *v* parametric plane is subdivided into elements of equal size. A set of geometric data points is associated to each element based on their position in the plane. This way, the coordinates of the geometric data points in high density regions are averaged, and the parametric surface cannot accurately mold the geometric data points in these regions. An increase in the number of control points can alleviate the problem, but does not solve it.

The RV polygonal mesh is particularly interesting, because the holes in the endocardial surface simply disappeared. This happened because the mapping algorithm generates a point distribution that expands in a manner to maximally fill the *u* − *v* parametric plane. The outer boundary is constrained but not the contours delimiting the holes. Thus, they are filled during the projection.

### 3.4. Polygonal Mesh Regularization

In order to accurately fit a parametric B-spline surface to the geometric data points, *Q*(*𝒱*) was regularized with respect to polygon area and edge length. To this end, we minimized a measure of variation in polygon deformation with respect to a reference polygon (7). See Appendix A for the details of this approach.

**Figure 4:**
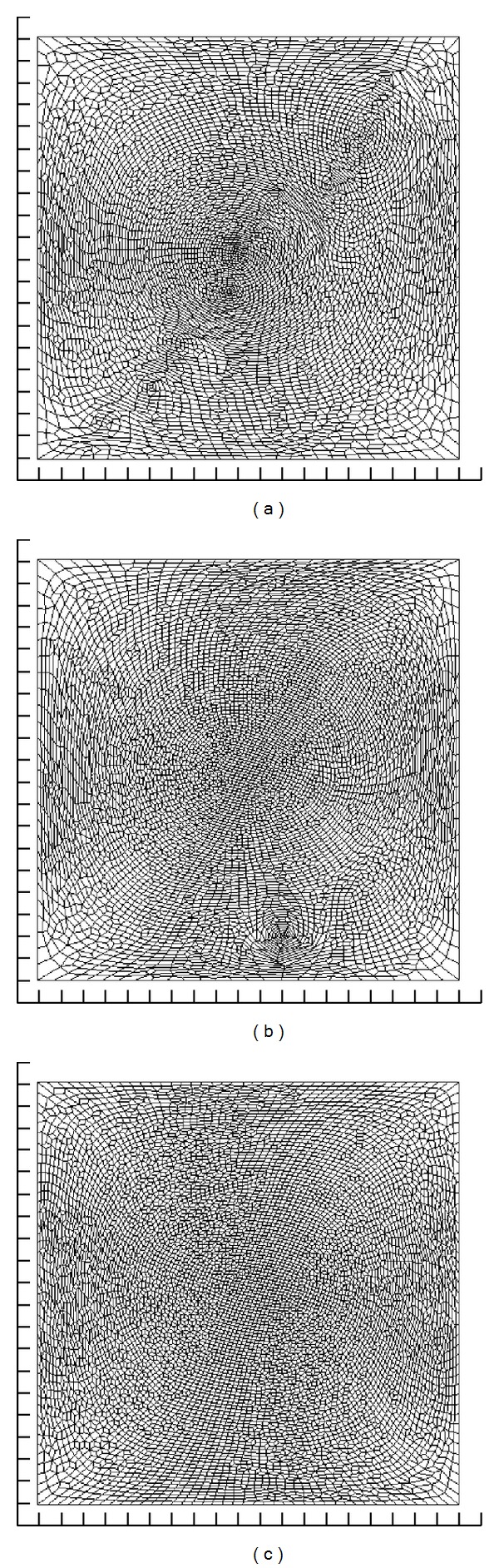
Polygonal meshes shown in Figure 3, regularized for polygons area and edges length.

**Figure 5:**
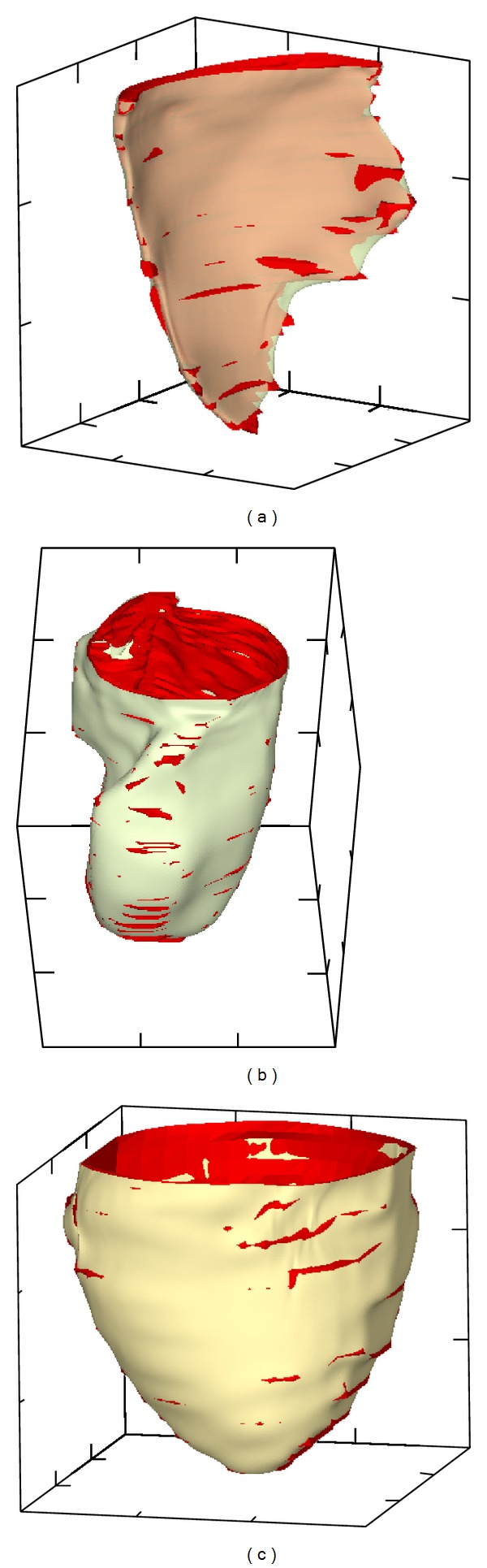
Parametric surfaces fitted to the *x* − *y* − *z* coordinates of the regularized polygonal meshes shown in Figure 4. (a) Right ventricular cavity; (b) left ventricular cavity; (c) epicardium. Gray: parametric surface. Red: Surface composed of triangles array generated from the brick wall texture of Figure 2.

**Figure 6:**
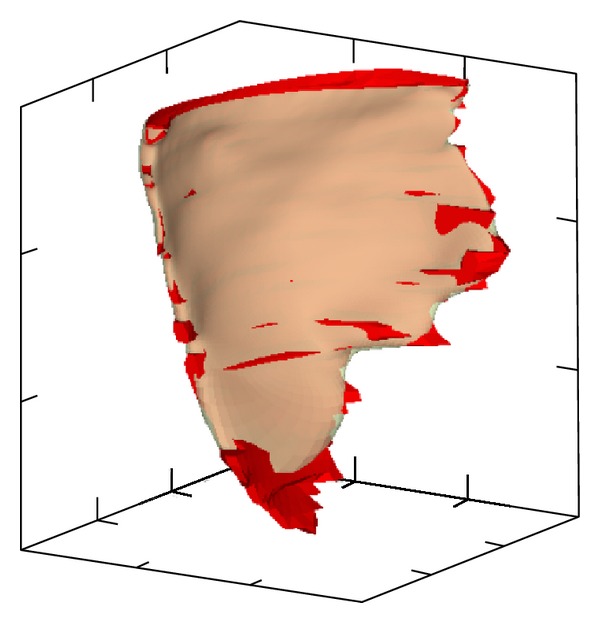
Parametric surface fitted to the *x* − *y* − *z* coordinates of the RV polygonal mesh before regularization, that is, Figure 3(a).

Figure 4 shows the polygonal mesh of Figure 3 after regularization. The regularized meshes are now quite uniform in polygon area and edge length. This is quite remarkable considering the initial variation in polygon density and the nonlinearity of the minimization problem. As a result of this regularization, the mapping is more conformal, and each element of the polygonal surface is given the same weight in the parametric surface fit (compare Figure 6 with Figure 5).

The polygon density of the regularized mesh is slightly higher along a diagonal in the RV surface. In order to refine regularization, one could reduce *ϵ* in the termination criteria ||**g**||_2_ < *ϵ*, though the increased number of iterations to solve the matrix system could dramatically increase the computation time (see Appendix A).

A note is in order regarding the computation time associated to with the regularization. Excluding regularization, the algorithm is quite fast; the projection and parametric surface fitting requires solving relatively simple matrix systems, both of which could be solved in the order of a second. On the other hand, the regularization is more demanding computationally since the procedure is iterative and the matrix expressing the minimization problem needs to be rebuilt at each iteration (see Appendix A). However, this task is not very time consuming if the algorithm is well implemented since most of the operations can be preprocessed.

### 3.5. Constrained Parametric Surface Fitting

**Figure 7:**
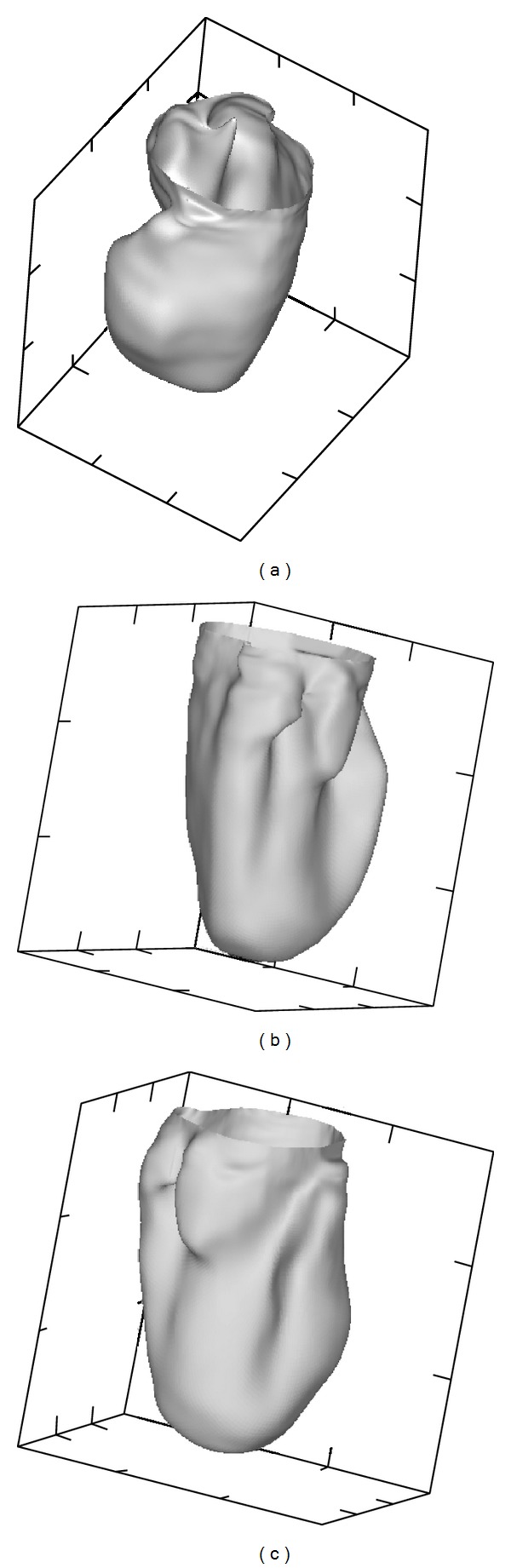
Multiple views of the parametric B-Spline surface fitted to the edge of the left ventricular cavity (Figure 2(b)).

**Figure 8:**
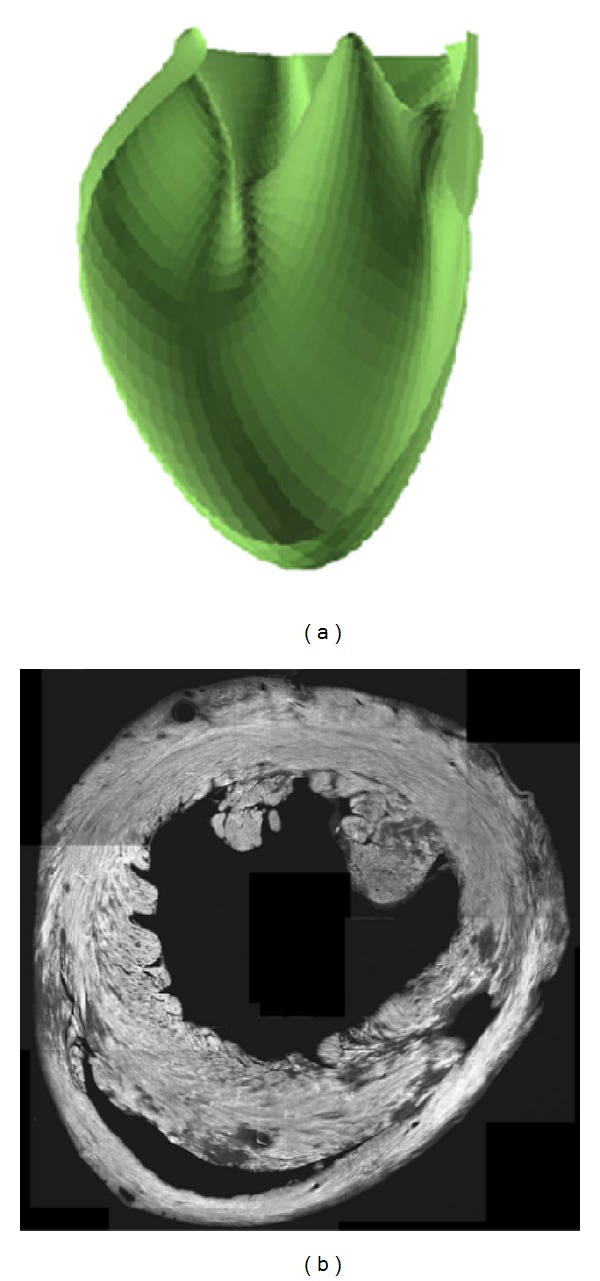
(a) Cut open view of the left ventricular cavity model, same as Figure 5(b). The view exposes the papillary muscle. (b) Laser scanning confocal microscopy image (4x enlargement) of a cross section of the same heart near the papillary muscle.

Figures 5–8 show the parametric surfaces fitted to the geometric data points. In each panel of Figure 5, the red surfaces are composed of a triangle array extracted from the brick wall texture of Figure 2. The grey surfaces are the codimension 2 B-splines fitted on the geometric data points of *Q*(*𝒱*) of Figure 4. The fit is performed according to the algorithm presented in the Methods.  Table 1 gives the parameter for the surface fits. All fits were performed by imposing a relatively low stiffness. This parameter is assigned by trial and error during visual inspection. It is convenient to proceed this way since, once the regularization completed, the fit is practically instantaneous.

As judged by Figure 5, the match between the parametric surfaces and the geometric data points is excellent. Convoluted surfaces, are accurately represented with only 9 × 9 elements, which is remarkable for the LV. Statistics on the distances between the parametric surface and the geometric data points are given in Table 2. Considering the number of polygons in each case (Table 1), this constitutes a dramatic reduction in the complexity of the representation and significantly impacts the computational labor of any task aiming to evaluate geometric information on this physiologic structure. Note the fit of the epicardial surface seems to be less accurate; that is, minimal, maximal, and averaged distances are larger than the one of the left and right ventricular cavities. However, also remark the variance is larger. The epicardial surface from CT is not as smooth as the surface of the cavities. There are local variations that could not be captured with the number of elements we used.

The right side of the RV parametric surface is smoother than the triangulated surface, but this result was desirable. The computerized tomography and the surface extraction are not highly accurate in this region since the surfaces nearly intersect one another. It was judged here that the smooth surface corrects some of these errors. Note that the holes in the RV polygonal surface (Figure 5) have been filled in the parametric surface without any manual interventions. There is a steep transition in the *x* − *y* − *z* coordinates near the holes, but they are smoothed out by the fit.

The fit of the LV surface is the most striking. The reproduction is excellent using only 9 × 9 elements in the parametric B-spline; this despite the fact that the surface is quite convoluted. The papillary muscle was accurately reproduced from its root in the endocardial surface all the way up to its tip near the aortic valve. Clearly the parametric surface fit is excellent. On average, the distance between a geometric data point and the surface is less than 8.0 *μ*m, 5.2 *μ*m, and 13.8 *μ*m for the surfaces bounding the RV cavity, LV cavity, and endocardium, respectively. In addition, we can observe the triangulated surface crossing in and out the parametric surface all around due to their proximity.

Figure 6 is a fit of the parametric surface of the RV polygonal mesh before its regularization (Figure 3). In this case, the apex is not well reproduced and important details are missed along the right side of the RV. In addition, the distance between the triangulated and parametric surfaces is larger. In this case, the triangulated and parametric surfaces do not cross one another as in Figure 5. Without regularization, the distance between the parametric surface and the geometric data can be quite large; that is, a convoluted geometry is smoothed out.

It is interesting to examine several views of the LV cavity in order to appreciate the accuracy of the reproduction, despite the convoluted nature of the geometry. Panel (a) of Figure 7 is a top view of the LV cavity exposing the papillary muscle. The surface is starting to close in its upper part because it is getting close to the heart valves. In panels (b) and (c), we have an outside view of several ridges, cavities, and protrusions.

Finally, Figure 8(a) shows a cut open view of the LV endocardial surface. The model includes protrusions, invagination, and a papillary muscle, while the surface remains smooth. Clearly, the representation is sufficiently flexible to generate a realistic model of the heart ventricles, with as few as 9 × 9 elements. We have not constrained the fit to the base, but could have done so. With the ability to constrain the fit on specific contours, we could have decomposed the geometric reconstruction to address more complex reconstruction problems.

Figure 8(b) is a confocal laser microscopy image of a cross-section of the heart near the root of the papillary muscle. The image was obtained at a 4x enlargement and after staining the cardiac fibers (see Methods). It illustrates how complex the subendocardial layer is. The cardiac fibers follow a feather-like pattern. They are oriented along the periphery in midmyocardium, but fan out as we move toward the periphery. The fibers enter the large protrusions along their long axis. In the papillary muscle we observe dots instead of lines, because at this level the fibers are oriented along the long axis of the papillary muscle. A detailed histology study by Robinson et al. [29] corroborates this result. We can also remark that the fibers are more separated in the protrusions and papillary muscle, thereby suggesting more collagen in these areas. Thus, clearly the ventricular walls have a complex structure. The interested reader is invited to consult Slamani et al. [19], Poddar et al. [20], and Subramanian et al. [30] for a high resolution three-dimensional reconstruction of cardiac fiber orientations based on laser scanning confocal microscopy data.

## 4. Discussion

### 4.1. Motivation for the Developed Technique

What motivated the developed technique was the specifications for geometric models employed to study problems in cardiac electrophysiology with computer simulations. Now that we illustrated a reconstruction with our method, we can elaborate on its application in investigative cardiology.

Several cardiac arrhythmias are initiated by an abnormal heart beat originating in the ventricles. The initiation site of these beats remains mysterious for a number of arrhythmias like congenital arrhythmias (e.g., Long QT syndrome), idiopathic ventricular tachycardias (IVT), cathecolaminergic ventricular tachycardias (CVT), and many others. It is likely that the triggering of abnormal beats occurs at sites displaying steep changes in electrical conduction because such sites are associated with local changes in propagation velocity, action potential duration (wave may pivot), and spatiotemporal distribution of intracellular calcium. All of which may favor wavefront and wavetail interactions, and in turn, the triggering of abnormal beats.

The root of large protrusions, the root of heart valves, the fascicles, the outflow tract, or any other convoluted region can display the above mentioned characteristics. There are two main reasons for this. First, these sites exhibit rapid changes in fiber orientation. For example, cardiac fibers are oriented along the tangent to the heart circumference in midmyocardium, but along the long axis of the protrusions, which implies a steep change in cardiac fiber orientation around the root of the protrusions. Second, in many instances these sites have higher collagen density because they play a structural role. Therefore, there is a need to investigate the role played by these sites in electrical conduction at a mesoscopic scale.

Cardiac modeling is definitively a good instrument for such investigation as it allows reconstructing electrical excitation from the ground up, and then studying the interplay between several contributing factors. The technique presented here offers the flexibility necessary for geometric heart reconstruction at the mesoscopic scale and all elements required for the formulation of an accurate electrical conduction model. Note that to perform simulations, one should add to the geometry a mathematical description of fiber and lamina orientations. An interesting technique to accomplish this, also based on *C*_2_ parametric representation, was documented by Bayer et al. [31].

### 4.2. Advantages and Limitations of Our Geometric Modeling Technique

We have presented a method to fit *C* ^2^ continuous parametric surfaces to scattered geometric data points on frontiers delimiting physiologic structures. Such surfaces meet the modeling needs stated above. So far the best way to generate them was through deformable models or level set methods. The approach we presented here is more direct and is likely to perform better. Briefly, once a polygonal surface is extracted from a segmented image, it is projected onto a parametric plane. The resulting polygonal mesh is regularized for polygon area and edge length. Then a parametric surface is fitted to the projected geometric data points, whose operation also assigns a unique parametric coordinate to each data point. The fit requires to adjust the control points of a codimension 2 B-spline in a manner to minimize the distance between the geometric data point and points on the surface with the same parametric coordinate. The computational load is relatively small. The projection and parametric surface fit necessitate solving a symmetric sparse matrix system with small bandwidth. When the polygonal surface is composed of quads, Nd × 9 coefficients, Nd: number of data points for the projection and Nb × 49 coefficients Nb: number of B-spline coefficients for the fit. These matrix systems can be solved in the order of seconds. The regularization is the time limiting step. It requires finding a function minimum. This is done with an iterative method, where each step includes the calculation of matrix coefficients and matrix-to-vector multiplications. The matrix in question is sparse and has a small bandwidth (dimension: Nd × 9 coefficients with quads).

Our results show it is possible to represent convoluted geometries in a compact manner with codimension 2 parametric B-splines. Specifically, a representation with 9 × 9 elements, or 121 control points, are sufficient to accurately describe the LV, including protrusions, invaginations, and even a papillary muscle.

The examination of advantages and disadvantages of this approach has to be contexted. A reconstruction technique can serve several purposes, for example, visualize a structure, detect features, measure areas and volumes, or investigate the role played by geometry with modeling and simulations. Obviously, each technique has advantages with respect to the targeted use. For visualization, when the image is not noisy and the contrast is high, thresholding with polygonal surface extraction is simple and fast, which meets the needs of this task. However, when the image is noisy the manual assistance required may make the technique too time consuming to be practical. A deformable model or a level set method would be more appropriate in this case. When there is too much noise, or the contrast is low, a technique replacing or adding to the function to minimize a comparison of each pixel's intensity with the averaged intensity of pixels inside and outside regions delimited by the moving boundary [14] is quite effective. However, this is done at the cost of a significant additional computational load. Still, this may be beneficial considering the manual labor involved by the alternative. In addition, for many tasks batch processing is quite acceptable. When one needs to perform measurements on the geometric reconstruction, as in oncology since the rate of uptake of a compound may depend on surface and dose on volume, then a mathematical representation amenable to such computation offers important advantages. Surfaces can be easily computed with triangle arrays. Deformable and level set methods can both provide such representation; however, the level set method necessitates an additional step to extract the surface. The calculation of volumes is more delicate. In this case a parametric representation dramatically facilitates this calculation. This also applies to the calculations required in modeling and simulations. Indeed, in this case the availability of *C*_2_ continuous surfaces is a great advantage since in addition to volume, precise calculations of curvature, distances on curved surfaces, and areas of curvilinear surfaces are needed. Such representation can be built with the deformable model or level set methods, but could significantly complicate the process. Alternatively, it could be included as a postprocessing operation, but as explained in the Introduction and Methods, it is not trivial to generate the *C*_2_ continuous parametric surface even when we know the frontier of the object in question. This is precisely the problem our technique addresses.

When the noise level is low and the contrast is high, which is the case for a large number of medical imaging modalities, the geometric reconstruction can be performed with thresholding and polygonal surface extraction followed with a fit of a *C*_2_ continuous surface like we did here. The computational load is minimal, results are obtained rapidly, and as illustrated here, accuracy is excellent. When the noise level is high or contrast is low, it would become advantageous to replace thresholding and polygonal surface extraction with a deformable model or a level set method. No need to say automaticity and robustness will come at the price of significant additional computational load.

Lastly, another limitation of our method is that the computational load rapidly increases with problem size. However, this can be addressed by decomposing the reconstruction problem. This means identifying, from the surface extraction, critical sections where the geometric model could be split, then fitting the border of these sections with *C*_2_ codimension 1 parametric B-splines to fit each portion separately by imposing the fitted surface to match the boundary curves. This could be further constrained by imposing a continuous surface normal orientation.

### 4.3. The Future of Medicine

With the use of imaging and genomic data, medicine is becoming more and more quantitative. This is to our benefice since it has significantly advanced diagnostics and the optimization of a number of therapeutic interventions. Nevertheless, there may be even more with the introduction of advanced computation; that is, modeling and simulations can provide unprecedented means to discover mechanisms of diseases.

As stated in “*motivation for the technique developed*”, we expect heart geometry and cardiac tissue microstructure to play an important role in the initiation of fatal arrhythmias. However, this role needs to be precisely discovered. Considering the unknown, it is appealing to approach this problem from the population scale, that is, finding in the population trends between symptoms or triggering conditions, with geometry and microstructure features. Considering the clinical data and the computational resources available to date, it is not unrealistic to attempt to tackle the problem this way.

First, it is common practice for any patient displaying recurrent episodes of tachycardia or syncopies to have a CT or MR or both scans. We could perform geometric heart reconstructions for this population, but this would require the reconstruction procedure to be automatic. Fortunately, such problem has been previously addressed. Few reconstructions are performed for cases displaying different geometries/microstructures. These reconstructions form an atlas which constitutes a basis of information that drives the automatic reconstructions. The approach is based on the* active shape modeling* introduced by Cootes et al. [32]. The variance of predefined markers coordinates are captured in a covariance matrix (our reconstruction method would facilitate this task). The first few eigenvectors corresponding to the largest eigenvalues of the covariance matrix provide axes to guide the constrained deformations. Indeed Frangi et al. [33] and Zheng et al. [34] built on this concept to develop elaborate computational infrastructure to reconstruct heart models automatically. Zheng et al. [34] even supplemented the technique with learning algorithms to generate classes of geometric models semiautomatically.

Once the reconstruction is performed at the population scale, features associated to electrophysiologic properties could be captured in several parameters. Then based on these parameters, model categories can be generated automatically with a classifier. In a subsequent step, correlations could be drawn between categories and conditions of initiation of arrhythmias. This specifies a number of parameters for the performance of elaborate simulations aiming at finding the causes triggering life threatening arrhythmias. The computational resources required for such an endeavor are considerable, but available at supercomputer centers. Such efforts could lead to the systematic discovery of mechanisms of arrhythmias, and in turn equip clinicians with new means to prevent life threatening arrhythmias and to optimize medical devices.

## Figures and Tables

**Figure 9:**
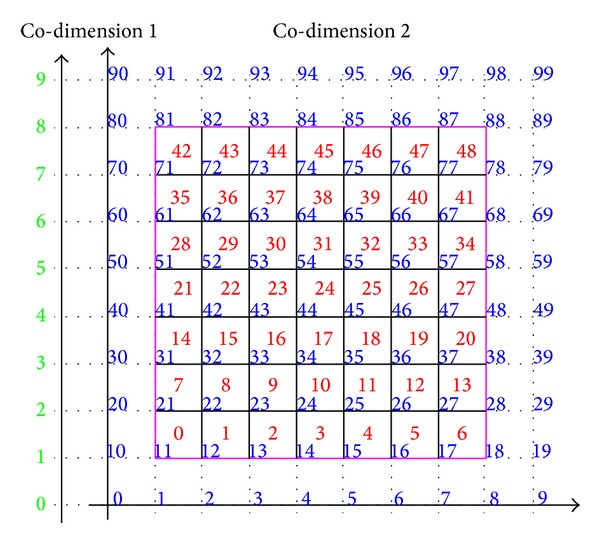
Parametric plane subdivided into 7 × 7 elements. Red labels: elements number. Blue labels: control points number. The border of the *u* − *v* parametric plane is in magenta.

**Table 1:** Parameters of the surface fitting. Number of polygons: number of polygons in the polygonal surfaces of Figure 2. *N*_*u*_, *N*_*v*_: number of elements along the *u* and *v* coordinates of the parametric plane. Stiffness: parameter *λ* in (13).

Surface	Number of polygons	*N*_*u*_	*N*_*v*_	Stiffness
Right ventricle	374,369	7	7	0.3
Left ventricle	377,863	9	9	0.2
Epicardium	1,013,085	11	11	0.1

**Table 2:** Min., Max., Avg. dist: minimal, maximal, and averaged distances, respectively. Distances refer to the distance between the geometric data points and the model surface, as calculated with the metric presented in Section 2. Rightmost column, variance of the distances. All distances and variance are in *μ*m.

Surface	Min. dist.	Max. dist.	Avg. dist.	Variance
Right ventricle	0.0334	25.9825	8.0064	15.5450
Left ventricle	0.0214	32.1468	5.2053	16.4575
Epicardium	0.1198	51.9895	13.8509	53.3334

## Appendix

### A. Numerical Solution of the Grid Regularization Equation

#### A.1. Quadrilateral Mesh

We minimize *F*(**u**, **v**)

(A.1) F(u,v)=∑e=0Ne−1∑i=01∑j=01∫Ωe[ri,j(e)]2  dΩ,R(e)=𝒥(e)−Adj{𝒥(e)}|𝒥(e)|,

where

(A.2) $$\begin{array}{c} {𝒥}^{\left(e\right)}=\left[\begin{array}{cc} U_{\xi }^{\left(e\right)} & U_{\eta }^{\left(e\right)}\\ V_{\xi }^{\left(e\right)} & V_{\eta }^{\left(e\right)} \end{array}\right],\;\;\text{Adj}\left\{𝒥\right\}=\left[\begin{array}{cc} V_{\eta }^{\left(e\right)} & \;\;-U_{\eta }^{\left(e\right)}\\ -V_{\xi }^{\left(e\right)} & U_{\xi }^{\left(e\right)} \end{array}\right],\\ {𝒥}^{\left(e\right)}=U_{\xi }^{\left(e\right)}V_{\eta }^{\left(e\right)}-U_{\eta }^{\left(e\right)}V_{\xi }^{\left(e\right)}, \end{array}$$

with respect to (**u**, **v**). Above the mapping functions are described with degree 1 Lagrange polynomials,

(A.3) U(e)(ξ,η)=∑i=03ui(e)Ψi(ξ,η),V(e)(ξ,η)=∑i=03vi(e)Ψi(ξ,η),

built with tensor product of one-dimensional Lagrange polynomials,

(A.4) Ψ(ξ,η)=ψ(ξ)⊗ψ(η),ψ(ξ)=[ψ0(ξ),ψ1(ξ)],ψ0(ξ)=1−ξ2,  ψ1(ξ)=ξ−12.

We find this minimum with a fixed point method. To this end, *F*(**u**, **v**) is approximated ($\tilde{F}(u,v)$) around (**u**_0_, **v**_0_) with a multidimensional quadratic

(A.5) F~(u,v) =12  (u−u0,v−v0)TH(u−u0,v−v0)  +  gT(u−u0,v−v0)+F(u0,v0)

admitting its minimum at

(A.6) $$\begin{array}{c} H\left[\begin{array}{c} u\\ v \end{array}\right]=g, \end{array}$$

where **H** and **g** are the Hessian (also called the Jacobian) and gradient of *F*(**u**, **v**) evaluated at (**u**_0_, **v**_0_).

The gradient **g** ^(*e*)^ ∈ *ℜ* ^8^ of an element “*e*”

(A.7) $$\begin{array}{c} g^{\left(e\right)}={\left(\frac{\partial F^{\left(e\right)}}{\partial u_{\alpha }^{\left(e\right)}},\frac{\partial F^{\left(e\right)}}{\partial v_{\alpha }^{\left(e\right)}}\right)}^{T},\;\alpha \in \left[0,3\right] \end{array}$$

is calculated taking the derivative of each component of **R** ^(*e*)^

(A.8) r0,0(e)=Uξ(e)  −  Vη(e)(Uξ(e)Vη(e)−Uη(e)Vξ(e)),r0,1(e)=Uη(e)  −  Uη(e)(Uξ(e)Vη(e)−Uη(e)Vξ(e)),r1,0(e)=Uξ(e)  −  Vξ(e)(Uξ(e)Vη(e)−Uη(e)Vξ(e)),r1,1(e)=Uξ(e)  −  Uξ(e)(Uξ(e)Vη(e)−Uη(e)Vξ(e)),

with respect to **u** ^(*e*)^, **v** ^(*e*)^, that is,

(A.9) $$\begin{array}{c} \frac{\partial F^{\left(e\right)}}{\partial u_{\alpha }^{\left(e\right)}}=\underset{i}{\sum }\underset{j}{\sum }\overset{1}{\underset{-1}{\int }}\overset{1}{\underset{-1}{\int }}2r_{i,j}^{\left(e\right)}\left(\frac{\partial r_{i,j}^{\left(e\right)}}{\partial u_{\alpha }^{\left(e\right)}}\right)d\xi \;d\eta \\ \frac{\partial F^{\left(e\right)}}{\partial v_{\alpha }^{\left(e\right)}}=\underset{i}{\sum }\underset{j}{\sum }\overset{1}{\underset{-1}{\int }}\overset{1}{\underset{-1}{\int }}2r_{i,j}^{\left(e\right)}\left(\frac{\partial r_{i,j}^{\left(e\right)}}{\partial v_{\alpha }^{\left(e\right)}}\right)d\xi \;d\eta , \end{array}$$

with

(A.10) $$\begin{array}{c} \frac{\partial r_{i,j}^{\left(e\right)}}{\partial u_{\alpha }^{\left(e\right)}}=\frac{\partial r_{i,j}^{\left(e\right)}}{\partial U_{\xi }^{\left(e\right)}}\frac{\partial \Psi_{\alpha }}{\partial \xi }+\frac{\partial r_{i,j}^{\left(e\right)}}{\partial U_{\eta }^{\left(e\right)}}\frac{\partial \Psi_{\alpha }}{\partial \eta },\\ \frac{\partial r_{i,j}^{\left(e\right)}}{\partial v_{\alpha }^{\left(e\right)}}=\frac{\partial r_{i,j}^{\left(e\right)}}{\partial V_{\xi }^{\left(e\right)}}\frac{\partial \Psi_{\alpha }}{\partial \xi }+\frac{\partial r_{i,j}^{\left(e\right)}}{\partial V_{\eta }^{\left(e\right)}}\frac{\partial \Psi_{\alpha }}{\partial \eta }. \end{array}$$

Once **g** ^(*e*)^ is evaluated, it is added to **g** with appropriate embedding based on *C*(*Q*).

Similarly, the Hessian **H** ^(*e*)^ ∈ *ℜ* ^8  ×  8^ of an element “*e*,”

(A.11) H(e)=[∂∂uβ(e)(∂F(e)∂uα(e))∂∂uβ(e)(∂F(e)∂vα(e))∂∂vβ(e)(∂F(e)∂uα(e))∂∂vβ(e)(∂F(e)∂vα(e))],   α,β∈[0,3],

is obtained taking the derivative of (A.9) with respect to **u** ^(*e*)^, **v** ^(*e*)^,

(A.12) ∂∂uβ(e)(∂F(e)∂uα(e)) =∑i∑j∫−11∫−112[(∂ri,j(e)∂uβ(e)∂ri,j(e)∂uα(e))     +  ri,j(e)(∂2ri,j(e)∂uβ(e)∂uα(e))]dξ  dη,∂∂uβ(e)(∂F(e)∂vα(e))=∑i∑j∫−11∫−112[(∂ri,j(e)∂uβ(e)∂ri,j(e)∂vα(e))      +ri,j(e)(∂2ri,j(e)∂uβ(e)∂vα(e))]dξ  dη,∂∂vβ(e)(∂F(e)∂uα(e))=[∂∂uβ(e)(∂F(e)∂vα(e))]T∂∂vβ(e)(∂F(e)∂vα(e))=∑i∑j∫−11∫−112[(∂ri,j(e)∂vβ(e)∂ri,j(e)∂vα(e))      +ri,j(e)(∂2ri,j(e)∂vβ(e)∂vα(e))]  dξ  dη

with

(A.13) ∂2ri,j(e)∂uβ(e)∂uα(e) =(∂Ψβ∂ξ∂Ψα∂ξ)∂2ri,j(e)∂Uξ(e)∂Uξ(e)  +(∂Ψβ∂η∂Ψα∂ξ)∂2ri,j(e)∂Uξ(e)∂Uη(e)  +(∂Ψβ∂ξ∂Ψα∂η)∂2ri,j(e)∂Uξ(e)∂Uη(e)  +(∂Ψβ∂η∂Ψα∂η)∂2ri,j(e)∂Uη(e)Uη(e),∂2ri,j(e)∂uβ(e)∂vα(e) =(∂Ψβ∂ξ∂Ψα∂ξ)∂2ri,j(e)∂Vξ(e)∂Uξ(e)  +(∂Ψβ∂η∂Ψα∂ξ)∂2ri,j(e)∂Vη(e)∂Uη(e)  +(∂Ψβ∂ξ∂Ψα∂η)∂2ri,j(e)∂Vξ(e)∂Uη(e)  +(∂Ψβ∂η∂Ψα∂η)∂2ri,j(e)∂Vη(e)Uη(e),∂2ri,j(e)∂vβ(e)∂vα(e) =(∂Ψβ∂ξ∂Ψα∂ξ)∂2ri,j(e)∂Vξ(e)∂Vξ(e)  +(∂Ψβ∂η∂Ψα∂ξ)∂2ri,j(e)∂Vξ(e)∂Vη(e)  +(∂Ψβ∂ξ∂Ψα∂η)∂2ri,j(e)∂Vξ(e)∂Vη(e)  +(∂Ψβ∂η∂Ψα∂η)∂2ri,j(e)∂Vη(e)Vη(e).

Once evaluated, **H** ^(*e*)^ is added to **H** with appropriate embedding as specified by *C*(*Q*). The derivatives with respect to *U*_*ξ*_^(*e*)^,  *U*_*η*_^(*e*)^,  *V*_*ξ*_^(*e*)^,  *V*_*η*_^(*e*)^ are known analytically from (A.8) and derivatives of  Ψ(*ξ*, *η*) with respect to *ξ*,  *η* are linear in *η*,  *ξ*.

All integrals are evaluated numerically with a Gauss-Legendre quadrature. We use 5 sampling points in each direction. The Vectors Ψ and their derivative are evaluated once and stored in memory. Then the solution proceeds as follows. Solve (A.6) for (**u** − **u**_0_, **v** − **v**_0_), (**u**, **v**)→(**u**_0_, **v**_0_), rebuild **g**, **H**, and keep iterating until ||**g**||_2_ < *ϵ*.

#### A.2. Triangular Mesh

The algorithm for a triangular mesh is obtained replacing the quad mapping functions with triangle mapping functions (triangle to triangle). With quads Ψ is built with a tensor product of elementary Lagrange polynomials. Instead with triangles,

(A.14) U(e)(ξ,η)=∑i=02ui(e)Ψi(ξ,η),V(e)(ξ,η)=∑i=02vi(e)Ψi(ξ,η),Ψ0(ξ,η)=1−(ξ+η),Ψ1(ξ,η)=ξ,Ψ2(ξ,η)=η

and one of the bound of integration in the *ξ* − *η* plane varies with one variable

(A.15) $$\begin{array}{c} \overset{\eta =1}{\underset{\eta =0}{\int }}\overset{\xi =1-\eta }{\underset{\xi =0}{\int }}f\left(\xi ,\eta \right)d\xi \;d\eta . \end{array}$$

This is quite conventional in finite element computation. The reader is referred to a finite element method textbook for more details on the subject. Indeed a good coverage of the subject can be found in Huebner et al. [35, Chapter 5]. Then embedding of the elementary vectors and matrices in the global matrix is done as specified by *C*(*T*). All the rest of the algorithm is the same as for the treatment of the quadrilateral mesh.

### B. Imposition of Boundary Conditions on Codimension 2 B-Splines

Consider the specific case illustrated in Figure 9. The parametric plane (*u* − *v*) is subdivided in 7 elements. The control points (blue) and elements (red) are numbered according to the directions of the *u* − *v* axes, *u*-ccordinate first then *v*. Two additional rows and columns of control points are added along the edges of the plane to ensure the B-Spline on boundary elements are represented with the same degrees of freedom as the other elements inside the plane. When assigning boundary conditions we fix values on these control points.

We clamp the edge *u* = 0 of the parametric plane with a contour *ℓ*(*v*). A codimension 1 B-spline is fitted to this contour. The parametric axis of this B-Spline is displayed on the left side, with elements and control points number in orange and green, respectively. This parametric axis is identical to the edge *u* = 0 of the *u* − *v* plane, that is, same length, same number of segments, each of them having the same length. The reader's attention is drawn to element 1 of the contour which corresponds to 7 in the plane. The part of the codimension 1 B-spline expansion contributing to this edge *ℓ* ^(1)^(*v*) is

(B.1) ℓ(1)(v)=ρ1b0(v¯)+ρ2b1(v¯)+ρ3b2(v¯)+ρ4b3(v¯),v¯=2v−v3+v2(v3−v2).

On any horizontal line, **b**(*u* = 0) take the values (1/6, 2/3, 1/6, 0). Thus the part of codimension 2 B-Spline expansion contributing to this edge is

(B.2) ℓ(1)(v) =16  ζ10b0(v¯)+23  ζ11b0(v¯)+16  ζ12b0(v¯)  +16  ζ20b1(v¯)+23  ζ21b1(v¯)+16  ζ22b1(v¯)  +16  ζ30b2(v¯)+23  ζ31b2(v¯)+16  ζ32b2(v¯)  +16  ζ40b3(v¯)+23  ζ41b3(v¯)+16  ζ42b3(v¯).

We Fix the coefficients on the boundary layer of the codimension 2 B-spline such that the coefficients factoring the same *b*_*i*_(*v*)*i* ∈ [0,3] in the codimension 1 and 2 B-splines are identical. This leads to

(B.3) $$\begin{array}{c} \left[\begin{array}{c} \frac{\zeta_{10}}{6}\\ \frac{\zeta_{20}}{6}\\ \frac{\zeta_{30}}{6}\\ \frac{\zeta_{40}}{6} \end{array}\right]=\left[\begin{array}{c} \rho_{1}\\ \rho_{2}\\ \rho_{3}\\ \rho_{4} \end{array}\right]-\left[\begin{array}{c} \frac{2}{3\;}\zeta_{11}+\frac{1}{6\;}\zeta_{12}\\ \frac{2}{3\;}\zeta_{21}+\frac{1}{6\;}\zeta_{22}\\ \frac{2}{3\;}\zeta_{31}+\frac{1}{6\;}\zeta_{32}\\ \frac{2}{3\;}\zeta_{41}+\frac{1}{6\;}\zeta_{42} \end{array}\right] \end{array}$$

a similar condition applies to the sides of the boundary elements along *u* = 0. An elementary B-spline on the codimension 1 contour has exactly 3 matching elementary B-splines on the codimension 2 surface, and their coefficients should be equal.

The boundary coefficients as determined by algebraic relation (B.3) for each boundary elements are introduced in the codimension 2 B-spline, eliminating the boundary layer from the codimension 2 expansion. The surface is fitted with this new system of equations. This condition guarantees the surface to exactly match the contour. As a rule we reintroduced the boundary layer coefficients in the original form of the codimension 2 expansion where each *ζ*_*s*_ factors a *B*_*s*_(*u*, *v*). This way all B-spline expansions always have the same format no matter what boundary conditions have been imposed to them.
